# Soluble Expression of hFGF19 without Fusion Protein through Synonymous Codon Substitutions and DsbC Co-Expression in *E. coli*

**DOI:** 10.3390/microorganisms8121942

**Published:** 2020-12-07

**Authors:** Hye-Ji Choi, Dae-Eun Cheong, Su-Kyoung Yoo, Jaehong Park, Dong-Hyun Lee, Geun-Joong Kim

**Affiliations:** Department of Biological Sciences and Research Center of Ecomimetics, College of Natural Sciences, Chonnam National University, Yongbong-ro, Buk-gu, Gwangju 61186, Korea; choihg1993@naver.com (H.-J.C.); decheong01@gmail.com (D.-E.C.); ysk_bio@naver.com (S.-K.Y.); applevd0827@hanmail.net (J.P.); donghyunlee73@chonnam.ac.kr (D.-H.L.)

**Keywords:** FGF19, DsbC, synonymous codon substitution, chaperon co-expression

## Abstract

Human fibroblast growth factor 19 (hFGF19) is a difficult-to-express protein that is frequently fused with another protein for soluble expression. However, residual amino acids after cleavage with protease represent one of the major problems in therapeutic protein development. Here, we introduced synonymous codon substitutions in the N-terminal region encoding sequence of *hFGF19* and co-expressed disulfide bond isomerase (ΔssDsbC) to functionally express hFGF19 without any fusion protein. Synonymous codon substitution significantly increased hFGF19 expression. Subsequent co-expression of ΔssDsbC with a selected variant of hFGF19 (scvhFGF19) further increased the proportion of soluble hFGF19 expression in *Escherichia coli* XL1-Blue. Both total and soluble scvhFGF19 expression increased remarkably in the alternative host, *E. coli* Origami 2 with mutated thioredoxin reductase and glutathione reductase. scvhFGF19 purification by anion exchange and heparin affinity chromatography resulted in a yield of 6.5 mg/L under normal induction conditions in flask culture. As such, a high cell density culture is expected to achieve an even higher yield. The biological activities of purified scvhFGF19 were assessed based on its ability to activate ERK1/2 signaling pathway in HepG2 hepatocarcinoma cells. In conclusion, the strategy described here may represent an efficient alternative process for the production of hFGF19 and/or related proteins.

## 1. Introduction

Fibroblast growth factor (FGF) is a representative growth factor that activates various signaling pathways by binding to fibroblast growth factor receptors (FGFRs) [1]. The amino acid sequences and tertiary structures of the 22 members of the human fibroblast growth factor (hFGF) family share similarities [2,3]. The hFGF family proteins are classified into seven groups based on phylogenetic analysis as well as intracrine, paracrine, and endocrine FGFs based on their mechanisms of action [4]. Endocrine hFGF19 belongs to the FGF19 subfamily comprising FGF19, 21, and 23, which have low binding affinity to heparin sulfate, allowing them to function as circulating molecules with effects on distant tissues [5]. Typically, hFGF19 is synthesized in the ileum in response to bile acid absorption and regulates various biological processes, such as the synthesis of cholesterol, glucose, lipid metabolism, and bile acid, thus representing a therapeutic agent for non-alcoholic fatty liver disease, insulin sensitivity, and reduction of adiposity [6,7].

In the crystal structure of hFGF19 (1PWA) [3], two disulfide bonds and long disordered structures in N- and C-terminal regions are observed; owing to these structural features, hFGF19 production in *Escherichia coli* is difficult. Recent advances in molecular biological tools, such as fusion proteins and genetically modified hosts, have facilitated functional expression of this difficult-to-express protein in the cytoplasm of *E. coli*, although the host environment is unfavorable for the formation of disulfide bonds [8]. Fusion proteins or tags are frequently used to facilitate soluble expression and purification of recombinant proteins. However, removal of these fusion proteins without any scar (amino acids remaining after digestion with protease) is difficult. In particular, it is vital for proteins to be free of scars when produced for fundamental research and clinical applications. Nevertheless, the functional expression of hFGF19 without any fusion tag or protein has not yet been reported in *E. coli*. 

We have previously reported that the solubility and expression of aggregation-prone and difficult-to-express proteins, respectively, are improved by synonymous codon substitutions in the N-terminal region encoding sequences of the corresponding genes [9,10]. Thus, in this study, we attempted to screen synonymous codon variants of hFGF19 (scvhFGF19). Although the expression level of scvhFGF19 was significantly improved, its soluble fraction was only marginally improved in *E. coli* XL1-Blue, the usual host used for expressing many proteins. We, therefore, inferred that improper disulfide bond formation in scvhFGF19 resulted in an insufficient expression of soluble protein. A chaperone was co-expressed with scvhFGF19, and the cytoplasmic redox condition was modulated to facilitate the formation of proper disulfide bonds, which increased the soluble expression of scvhFGF19. The scvhFGF19 thus obtained was purified using anion exchange and heparin affinity chromatography successively. The purified scvhFGF19 fully activated ERK1/2 signaling pathway, which is mediated through interactions of FGF19 with FGFR. To our knowledge, this is the first report regarding the expression and purification of hFGF19 without a fusion partner in *E. coli*, and this strategy may facilitate an improved expression quality of similar difficult-to-express FGFs for fundamental research and therapeutic applications.

## 2. Materials and Methods 

### 2.1. Bacterial Strains and Chemicals

*Escherichia coli* XL1-Blue (Δ(mcrA)183 Δ(mcrCB-hsdSMR-mrr)173 endA1 supE44 thi-1 recA1 gyrA96 relA1 lac (F′ proAB lacIqZΔM15 Tn10 (Tet^r^))), Origami2 (Δ(ara-leu)7697 ΔlacX74 ΔphoA PvuII phoR araD139 ahpC galE galK rpsL F′ (lac+ lacIq pro) gor522::Tn10 trxB (Str^R^, Tet^R^)), and Origami (DE3) (Δ(ara-leu)7697 ΔlacX74 ΔphoA PvuII phoR araD139 ahpC galE galK rpsL F′ (lac+ lacIq pro) (DE3) gor522::Tn10 trxB (Kan^R^, Str^R^, Tet^R^)) were used as hosts to construct randomly substituted synonymous codon libraries and express the screened codon variants of scvhFGF19. The genomic DNA of *E. coli* W3110 was isolated and used as a template to amplify the *dsbC* gene. pET24a_hFGF19 was kindly provided by Dr. Lee Jung-Hyun (Korea institute of Ocean Science & Technology, Busan, Korea) and used as a template to amplify the *hFGF19* gene by PCR with specifically designed primers having synonymous codons at the 5′-terminal region [9]. pSCT5-mCherry [10] was used for constructing the synonymous codon library. pACYCDuet1 (New England Biolabs, Hitchin, UK) and pQE80L (Qiagen, Hilden, Germany) were used as backbones for the plasmid constructs containing the *dsbC* and *scvhFGF19* genes, respectively, for co-expression in the cytoplasm of *E. coli*. The primers used in this study are listed in Table 1. Recombinant FGF19 was purchased from ProSpec-Tany TechnoGene (Israel) and used as positive control for the biological assay of purified scvhFGF19.

### 2.2. Library Construction and Screening of Synonymous Codon Variants

Synonymous codon libraries of *hFGF19* were constructed as previously described [9]. Briefly, the *hFGF19* gene was amplified by PCR with a set of primers using Phusion polymerase (New England Biolabs, Ipswich, MA, USA), followed by its incorporation into the vector pSCT5_mCherry, which was digested with *Spe*Ⅰ and *Hind*Ⅲ or *Spe*Ⅰ and *Bam*HⅠ according to standard cloning procedures, generating pSCT5_hFGF19 as a control and pSCT5_scvhFGF19-mCherry as a library for screening codon variants, respectively. The set of primers used was either pSCT5_FGF19-mC-fw and pSCT5_FGF19-mC-rv or pSCT5_scvFGF19-mC-fw and pSCT5_FGF19-mC-rv. The resulting recombinant plasmids were transformed into *E. coli* XL1-Blue for cloning and library construction. From the synonymous codon libraries, scvhFGF19-mCherry codon variants with improved expression were screened based on the enhanced fluorescence of red fluorescent mCherry.

### 2.3. Analysis of Expression Pattern of mCherry-Fused scvhFGF19 Variants

To analyze the expression patterns of the selected synonymous codon variants, recombinant *E. coli* cells expressing pSCT5_scvFGF19-mCherry variants were streaked onto Luria Bertani (LB) agar plates supplemented with 100 µg/mL ampicillin and then grown at 37 °C. Each of the resulting single colonies on LB agar was separately seeded into 4 mL LB broth containing 100 μg/mL ampicillin and incubated at 37 °C on an orbital shaker (220 rpm; shaking diameter, 5 cm). After overnight cultivation, 1% (*v/v*) of the culture broth was inoculated into fresh LB broth and cultured under the same conditions. After reaching an optical density of about 0.5–0.6 at 600 nm (OD_600_), 4 μL of 200 mM isopropyl β-D-1-thiogalactopyranoside (IPTG, final concentration of 0.2 mM) was added to the culture, which was further incubated for 3 h under the same conditions to induce protein expression. The resulting culture was harvested by centrifugation at 16,100× *g*, 4 °C. Harvested cells were resuspended (adjusted to an OD_600_ of about 2.0) in 200 μL of 20 mM sodium phosphate buffer (pH 7.4) and then sonicated for 4 min 30 s (2 s pulse and 8 s break for cooling) at 4 °C to disrupt the cells. The insoluble aggregates in cell lysates were removed by centrifugation at 16,100× *g* for 30 min at 4 °C. Total protein and soluble protein samples were obtained from the cell lysates and supernatant after centrifugation, respectively. Aliquots of total and soluble protein were mixed with sample loading buffer (0.225 M Tris-HCl pH 6.8, 50% glycerol, 5% SDS, 0.005 bromophenol blue, and 0.25 M DTT) at a 1:4 ratio and boiled for 15 min before resolving by SDS-PAGE on 12% gels. After electrophoresis, gels were stained with Coomassie blue staining solution.

### 2.4. Analysis of the Expression Pattern of scvhFGF19 Variants without Fusion Partner

After the expression analysis of mCherry-fused scvhFGF19, the selected variants were amplified by PCR with the primers pSCT5_scvFGF19-fw and pSCT5_scvFGF19-rv, followed by digestion with *Spe*Ⅰ and *Hind*Ⅲ, and then subcloned into pSCT5 plasmid digested with the same restriction enzymes to remove the gene encoding the fusion partner mCherry. Subsequently, the expression patterns of the scvhFGF19 variants without fused mCherry were reanalyzed in the same manner as those of the mCherry-fused scvhFGF19 variants.

### 2.5. Construction of Plasmids for Co-Expression of DsbC and scvhFGF19

**Figure 1:**
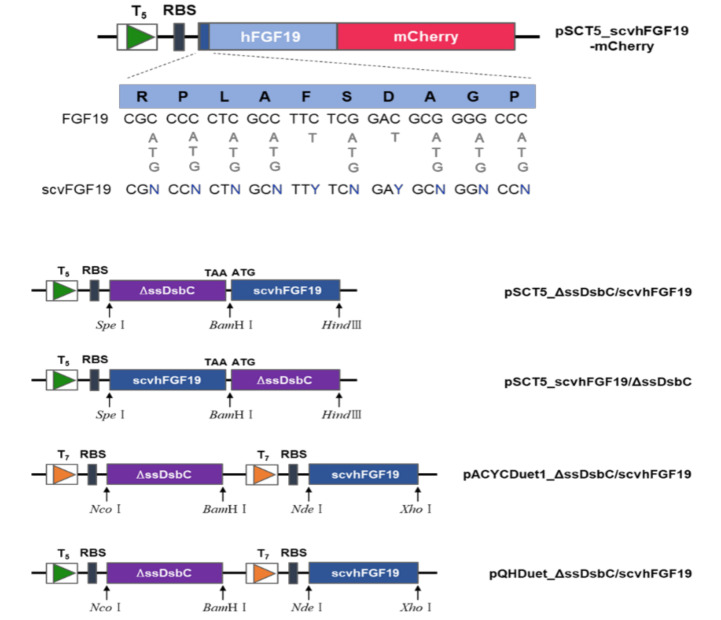
Schematic representation of the plasmids constructed in this study for the screening and expression of scvhFGF19. Upper panel shows a genetic map of the hFGF19-mCherry fusion protein and wobble sequences introduced into the N-terminal region of hFGF19 for codon variant library construction. Lower panel reveals schematic maps of the plasmids used for the co-expression of scvhFGF19 with ΔssDsbC under single, two identical, and two different promoters.

To co-express the screened scvhFGF19 and the chaperone DsbC in *E. coli*, three constructs were prepared. First, to express the *dsbC* and *scvhFGF19* genes as a single polycistronic mRNA in *E. coli* cytoplasm, *ΔssdsbC* gene encoding DsbC without the signal peptide from 2 to 20 amino acids was amplified from the genomic DNA of *E. coli* W3110 by PCR using either pSCT5_ΔssDsbC-fw and pSCT5_ΔssDsbC-rv or pSCT5_FGF19/ΔssDsbC-fw and pSCT5_FGF19/ΔssDsbC-rv, and the resulting DNA fragments were digested with the restriction enzymes *Spe*Ⅰ and *Bam*HⅠ or *Bam*HⅠ and *Hind*Ⅲ, respectively, followed by cloning into pSCT5_scvhFGF19 digested with the same enzymes. The two constructs, pSCT5_ΔssDsbC/scvhFGF19 and pSCT5_scvhFGF19/ΔssDsbC, were generated by incorporating the gene encoding ΔssDsbC into the upstream and downstream regions of *scvhFGF19*, respectively (Figure 1). 

Second, to independently express each gene under the same promoter T7, the *ΔssdsbC* gene was amplified by PCR using the primers pACYCDuet1_ΔssDsbC-fw and pACYCDuet1_ΔssDsbC-rv, while *scvhFGF19* was amplified using pACYDuet1_scvhFGF19-fw and pACYCDeut1_scvhFGF19-rv. The resulting *ΔssdsbC* gene was digested with *Nco*Ⅰ and *Bam*HⅠ, followed by incorporation into pACYCDuet1 vector digested with the same enzymes, generating pACYCDuet1_DsbC. The *scvhFGF19* gene was digested with *Nde*Ⅰ and *Xho*Ⅰ, followed by incorporation into pACYCDuet1_DsbC vector digested with the same enzymes, resulting in the construct pACYCDuet1_ΔssDsbC/scvhFGF19 (Figure 1).

Third, to independently express each gene under different promoters (T5 and T7), the DNA sequence between *ΔssDsbC* and *scvhFGF19* of pACYCDuet1_ΔssDsbC/scvhFGF19 was amplified by PCR with the primers pQHDuet_InfuΔssDsbC-fw and pQHDuet_InfuΔssDsbC-rv, then recombined with the DNA fragment amplified from the plasmid pQE80L with the primers pQE80L V-fw and pQE80L V-rv using an In-Fusion cloning Kit (Takara Bio, Kusatsu, Japan), and finally subcloned into the vector pQE80L. The resulting construct was named pQHDuet_ΔssDsbC/scvhFGF19 (Figure 1). 

### 2.6. Analysis of scvhFGF19 Expression Patterns Due to Co-Expression of ΔssDsbC in E. coli Cytoplasm

The four plasmid constructs were transformed into *E. coli* XL1-Blue, Origami 2, and Origami (DE3) by incubating competent cells with each plasmid under the general conditions for heat shock treatment. Using harvested cells after the culture of the transformed cells, total and soluble fractions of the expressed proteins were prepared as described in the analysis of the expression pattern of mCherry-fused scvhFGF19 variants. The expression patterns of scvhFGF19 were analyzed using SDS-PAGE and Western blotting. For Western blot analysis, the proteins separated by SDS-PAGE (15%) were transferred into a nitrocellulose membrane (GE healthcare, Chicago, IL, USA) using the Power Blotter-Semi-dry transfer system (ThermoFisher scientific, Waltham, MA, USA) according to the manufacturer’s instructions. The membrane was then blocked using 5% skim milk for 1 h, followed by overnight probing at 4 °C with anti-human FGF19 monoclonal antibody (1:5000, R&D Systems, Minneapolis, MN, USA). Next, the membrane was incubated with horseradish peroxidase-linked goat anti-mouse immunoglobulin G (1:5000, Enzo Life Sciences, Ann Arbor, MI, USA) at room temperature for 1 h. The proteins on the membrane were visualized using the ECL kit detection system (Bio-Rad, Hercules, CA, USA).

### 2.7. Purification of scvhFGF19

For the purification of the selected variant of scvhFGF19, recombinant *E. coli* Origami (DE3) cells harboring pQHDuet_ΔssDsbC/scvhFGF19 were streaked onto Luria Bertani (LB) agar plates supplemented with 100 µg/mL ampicillin and then grown at 37 °C. Single colonies on LB agar were individually seeded into 4 mL LB broth containing 100 μg/mL ampicillin and incubated at 37 °C and 220 rpm until the cultures reached early stationary phase. Then, a 1 L flask containing 200 mL of LB broth with the same antibiotics was inoculated with 1% (*v/v*) of the seed culture and incubated at 220 rpm and 37 °C until OD_600_ of the culture reached 0.6. Subsequently, the cells were induced with 0.2 mM IPTG and further incubated at 30 °C for 3 h, with constant shaking at 240 rpm. Finally, the cultured cells were harvested by centrifugation at 4 °C and 10,000× *g* for 10 min. 

Based on the theoretical isoelectric point and heparin affinity of hFGF19, Q HP anion exchange chromatography and a heparin HP column were successively used for purifying scvhFGF19. Considering the theoretical isoelectric point (6.5), the Q HP column (5 mL; GE Healthcare, Chicago, IL, USA) was selected for primary purification. The harvested cells were resuspended in the binding buffer (20 mM sodium phosphate buffer, pH 7.3) and disrupted by sonication; insoluble aggregates were removed by centrifugation at 20,000× *g* and 4 °C for 30 min. The resulting supernatant was loaded onto the Q HP column that had been pre-equilibrated with binding buffer. After binding, the column was completely washed with washing buffer (20 mM sodium phosphate buffer, pH 7.3), and then eluted by using a linear gradient of elution buffer (20 mM sodium phosphate buffer, 1 M NaCl, pH 7.3). The purity of the eluted protein in each fraction was analyzed by SDS-PAGE. The fractions containing scvhFGF19 were collected and diluted five times with a dilution buffer (20 mM sodium phosphate, pH 6.5) before loading onto the heparin column pretreated with the dilution buffer. The column was then completely washed with the same buffer, then eluted using a linear gradient of 0.1–1 M NaCl. The purity of the protein in eluted fractions was determined by SDS-PAGE. 

### 2.8. Endotoxin Removal

Endotoxins in eluted protein solutions were removed using a high capacity endotoxin removal spin column (ThermoFisher Scientific, Waltham, MA, USA) according to the manufacturer’s instructions, and its concentration in protein solution was determined using the LAL endotoxin assay kit (ThermoFisher Scientific, Waltham, MA, USA). There was no difference between the resulting protein and control buffer solution. The concentration of the purified recombinant scvhFGF19 was determined using Bradford assay with bovine serum albumin (BSA) as a standard.

### 2.9. Activity Assay of scvhFGF19

HepG2 hepatocellular carcinoma cells were cultured in DMEM media containing 10% fetal bovine serum (FBS), 100 U/mL penicillin, and 100 μg/mL streptomycin until the mid-logarithmic phase, then transferred to a 96-well plate (5 × 103/well). After incubation at 37 °C for 24 h, the cells were starved in DEME supplemented with 0.4% FBS for 24 h prior to treatment of the purified scvhFGF19. HepG2 cells were treated with 5 μg/mL scvhFGF19 at different concentrations for 15 min (or indicated time), followed by cell disruption with lysis buffer (25 mM Tris-HCl pH 7.5, 150 mM NaCl, 1 mM EDTA, 1% NP-40, and 5% glycerol) containing protease inhibitor cocktail (Quartett GmbH, Germany) and phosphatase inhibitor cocktail (AG Scientific, San Diego, CA, USA). Following centrifugation at 12,000× *g* and 4 °C for 20 min, total protein concentration of the supernatant was measured using the BCA kit (ThermoFisher Scientific, Waltham, MA, USA) and separated by SDS-PAGE. From the lysates, biological activity of scvhFGF19 was analyzed by immunoblotting with antibodies against Erk1/2 (Cell Signaling Technology, Danvers, MA, USA), phosphorylated Erk1/2 (Cell Signaling Technology), and α-tubulin (Sigma-Aldrich, St. Louis, MO, USA). 

## 3. Results

### 3.1. Expression of scvhFGF19 Screened from Synonymous Codon Variant Library

**Figure 2:**
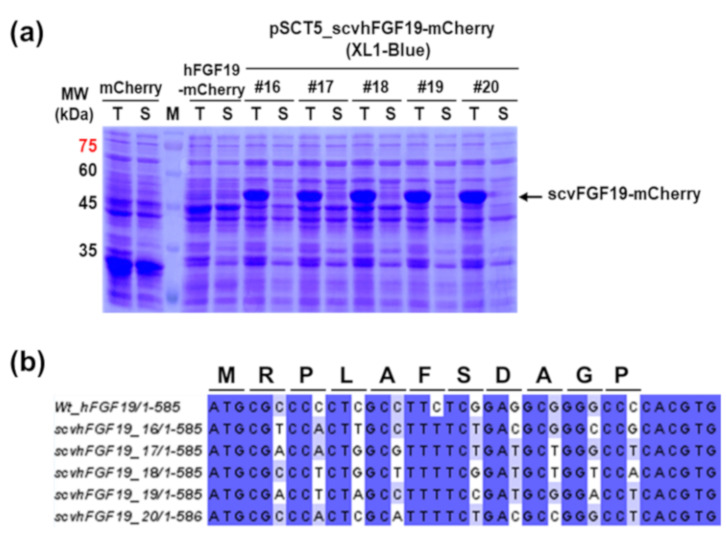
SDS-PAGE analysis and DNA sequence alignment of the selected codon variant scvhFGF19: (**a**) Analysis of the expression of the selected variant scvhFGF19-mCherry fusion proteins. MW: protein size marker; T and S: total and soluble fraction; mCherry: expressed mCherry as a control; hFGF19: mCherry-fused with wild type hFGF19; 16–20: screened codon variant scvhFGF19-mCherry. (**b**) Alignment of DNA sequence of the screened scvhFGF19 with substituted synonymous codons in the N-terminal region.

To express hFGF19 without any change in the amino acid sequence, codons encoding the first ten amino acids except for ATG were randomly substituted with synonymous codons as described previously [10]. From synonymous codon variant (scv) libraries consisting of approximately 2.8 × 10^4^ independent clones, 20 scvhFGF19-mCherry-fused variants were preliminarily screened based on red fluorescence intensity, using fused mCherry as a reporter (Figure S1). Among them, five mCherry-fused variants (Figure 2a) were selected after SDS-PAGE and Western blot analysis based on their relatively high expression level and soluble fraction ratio. Although the results from the DNA sequence analysis were insufficient to address the different expression levels of the selected variants, the corresponding sequence to each variant showed that codons encoding amino acids in N-terminal region were randomly replaced with synonymous codons (Figure 2b). 

**Figure 3:**
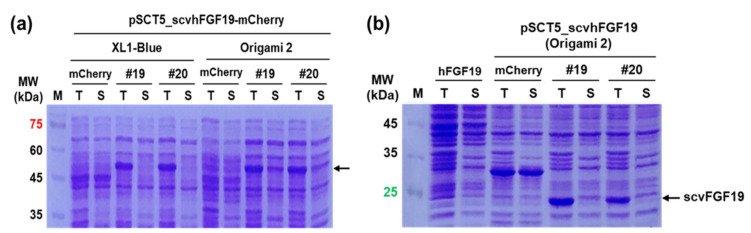
Analyses of the expression of the selected codon variants 19 and 20 in *E. coli* XL1-Blue and Origami 2. (**a**) Expression patterns of the selected codon variants with mCherry fusion in *E. coli* XL1-Blue and Origami 2. mCherry: expressed mCherry as a control; 19–20: the selected codon variants with mCherry fusion. (**b**) Expression patterns of the two selected codon variants without the fusion partner mCherry in *E. coli* Ogriami 2. mCherry: expressed mCherry as a control; hFGF19: expression of wild type hFGF19 without mCherry as a control; 19–20: expression of codon variants 19 and 20 without mCherry.

scvhFGF19-mCherry-expressing cells harvested from culture broth exhibited light pink color under visible light due to the high expression level of fused-mCherry. In contrast, cells expressing the wild type hFGF19-mCherry had no color. As expected, the distinct protein bands corresponding to the wild type hFGF19 and its fusion protein hFGF19-mCherry, respectively, were not detected in SDS-PAGE (Figure 2). In contrast, the protein bands corresponding to the selected scvhFGF19 variants were clearly detected. However, almost all expressed proteins formed an insoluble aggregate as an inclusion body (Figure 2a), although mCherry emitted red fluorescence in the screening step; these results were inconsistent with our previous reports [10,11]. We thus speculated that marginal soluble expression of scvhFGF19 was probably associated with structural features such as the two internal disulfide bonds and the long, disordered region at the N- and C-terminus [12]. As the reducing environment of the *E. coli* cytoplasm is unfavorable for the formation of disulfide bonds, correct folding of scvhFGF19 may be impaired. To test this speculation, we used *E. coli* Origami 2 as an alternative host strain for the expression of two codon variants, 19 and 20, in which the expression levels were relatively reproducible regardless of mCherry fusion among the five selected variants (Figure 3). Origami 2 strain is a K-12 derivative that resembles XL1-Blue and has mutations in both thioredoxin reductase (*trxB*) and glutathione reductase (*gor*) genes, thus promoting disulfide bond formation in the *E. coli* cytoplasm. However, only a slight increase was observed in the expression levels of the soluble fraction of scvhFGF19-mCherry when *E. coli* Origami 2 was used as a host (Figure 3a). These trends were also observed when the fusion partner mCherry was removed from the construct (Figure 3b), suggesting that either the innate solubilities of the selected scvhFGF19 variants were not improved or these variants were misfolded in the cytoplasm due to incorrect disulfide bond formation. To enhance the soluble expression of scvhFGF19 without the reporter mCherry, codon variant 19 was used as a model for further experiments due to its relatively higher solubility compared with that of codon variant 20. When required, codon variant 20 was also used as a control.

### 3.2. Enhanced Soluble Expression of scvhFGF19 Due to Co-Expression of DsbC in E. coli Cytoplasm

Disulfide bond formation in *E. coli* is mainly catalyzed by DsbC or the DsbA/B complex in the periplasmic space. To support disulfide bond formation in the cytoplasm, we employed the disulfide bond isomerase DsbC, which reduces two active site cysteines through a pathway involving the cytoplasmic proteins thioredoxin reductase, thioredoxin, and cytoplasmic membrane protein DsbD, thereby assisting the correct folding of proteins with multiple disulfide bonds even when expressed in the cytoplasm [13,14]. To co-express DsbC and the codon variant 19 in the cytoplasm of *E. coli*, a *dsbC* gene without a signal sequence (2-22 aa) (*∆ssdsbC)* was incorporated downstream or upstream of the codon variant 19 in pSCT5_scvhFGF19 and transcribed in a polycistronic manner under the control of a single T5 promoter. The expressed codon variant 19 was quantitatively analyzed by Western blotting because the sizes of the proteins scvhFGF19 (22 kDa) and ∆ssDsbC (23.5 kDa) were indistinct in SDS-PAGE. 

**Figure 4:**
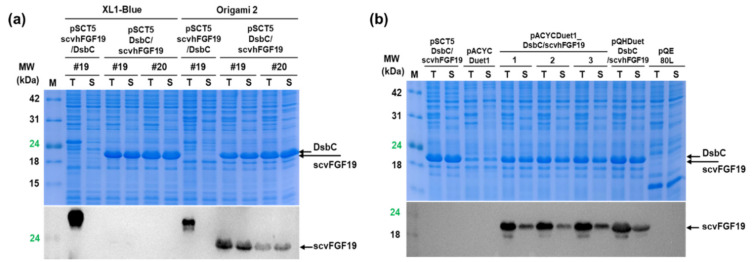
Expression analysis of DsbC co-expressed codon variant hFGF19. (**a**) Codon variant hFGF19 and DsbC were independently co-expressed under a single promoter. In this experiment, two constructs were used: scvhFGF19/ΔssDsbC and ΔssDsbC/scvhFGF19 (denote the genetically expressed order). Two hosts—*E. coli* XL1-Blue and Origami 2—were used to compare the expression patterns under different reduction conditions of the cytoplasm. The lower panel shows a Western blotting result of the expressed codon variant 19. For clearer comparison, codon variant 20 was used as a positive control. (**b**) Co-expression of codon variant 19 and DsbC under two identical and different promoters in *E. coli* Origami 2 and Origami (DE3) were analyzed. pACYCDuet1 and pQE80L empty vectors were used as negative controls. Lanes 1–3: repeated experimental results of the clones harboring pACYCDuet1_ΔssDsbC/scvhFGF19 under two identical T7 promoters; pQHDuet_ΔssDsbC/scvhFGF19: the expression of DsbC and scvhFGF19 under T5 and T7 promoters, respectively. Arrows indicate the protein size corresponding to their prediction molecular weight: DsbC (23.5 kDa), scvhFGF19 (22 kDa). SDS-PAGE results for DsbC and scvhFGF19; overlapped bands were obtained.

In both cases, although DsbC co-expression significantly increased the proportion of the soluble codon variant 19 in *E. coli* Origami 2 cytoplasm, the total expression level of this variant was approximately two times lower than that without ∆ssDsbC. Interestingly, ∆ssDsbC incorporation upstream of the codon variant 19, which resulted in earlier expression of the protein encoded by it, increased the expression level of soluble scvhFGF compared with that due to its incorporation in the downstream region of codon variant 19, as shown by the SDS-PAGE and Western blotting results of the expressed proteins when XL1-Blue and Origami 2 were used as two hosts (Figure 4a). The DNA sequences and maps for the physical locations of codon variant 19 and DsbC in the pSCT5_scvhFGF19/DsbC plasmid completely matched with the intended design strategy. Nevertheless, in the case of later expression of ∆ssDsbC, due to the incorporation of the gene encoding it in the downstream region of the codon variant 19, anti-hFGF19 monoclonal antibody mainly bound to an ambiguous protein with a higher molecular weight than the expected size of scvhFGF19 owing to unknown reasons. Based on these results, we determined the beneficial effects of DsbC co-expression as a chaperone and use of Origami 2 as a host for the soluble expression of scvhFGF19 without fusion partners, although the expression level required further improvements for practical applications. 

### 3.3. Improved Total Expression of scvhFGF19

We constructed pACYCADuet1_DsbC/scvhFGF19 and pQHDuet_DsbC/scvhFGF19, wherein ∆ssDsbC and scvhFGF19 were independently expressed under two identical T7 promoters and T5 and T7 promoters, respectively (see Figure 1). In the former case, when ∆ssDsbC and scvhFGF19 were separately expressed under the control of two independent T7 promoters in pACYCADuet1_DsbC/scvhFGF19, the expression level of scvhFGF19 increased by about 1.5-fold and was recovered to approximately 90% of that under T5 promoter in pSCT5_scvhFGF19 (Figure 4b). However, total and soluble expression levels of the codon variant 19 in pACYCADuet1_DsbC/scvhFGF19 fluctuated in the repeat experiments. In the latter case, when ∆ssDsbC and scvhFGF19 were expressed under T5 and T7 promoters, respectively, in pQHDuet_DsbC/scvhFGF19, the expression level of the codon variant 19 increased to approximately 1.3–1.5-fold compared with that of pACYCADuet1_DsbC/scvhFGF19 and showed a high reproducible result. Thus, the clone harboring the construct pQHDuet_DsbC/scvhFGF19 was used for the purification of scvhFGF19.

### 3.4. Purification of scvhFGF19

**Figure 5:**
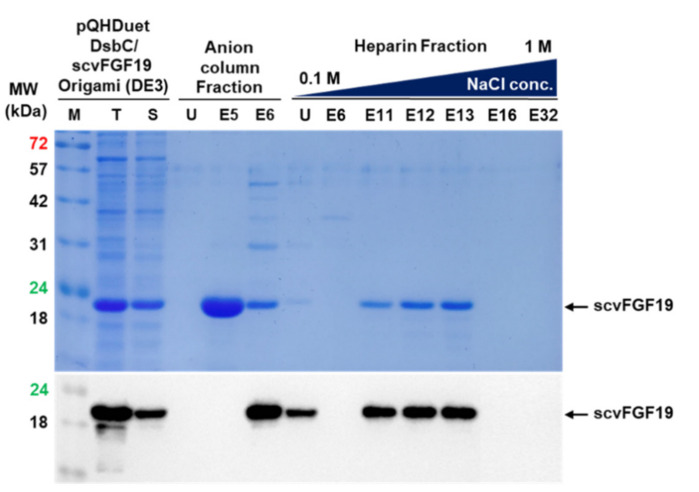
Purification of the codon variant 19 through the successive application of anion exchange and heparin affinity chromatography. M: protein size marker; T and S: total and soluble fraction; U: flow through; E5, E6, E10, E16, E17, E18, and E24: fraction number.

Recombinant scvhFGF19 was purified from *E. coli* Origami (DE3) cell lysate using a two-step purification procedure, consisting of anion exchange and heparin affinity column chromatography, according to the experimental procedure described in the methods section. In the first step, the fraction containing scvhFGF19 was eluted from the column using the concentration gradient corresponding to 160 to 220 mM NaCl. The second step, which involved purification using heparin column, reproducibly eluted scvhFGF19 from the column using salt concentrations of 400–500 mM NaCl (Figure S2). Figure 5 shows that ∆ssDsbC was separated from scvhFGF19 after anion exchange column chromatography, although other contaminants remained. After heparin affinity column chromatography, more than 90% purity of scvhFGF19 was reproducibly obtained (Figure S2). The final yield of purified scvhFGF19 from Origami (DE3) strain was approximately 6.5 mg/L of culture broth.

### 3.5. Biological Activity of Purified scvhFGF19

**Figure 6:**
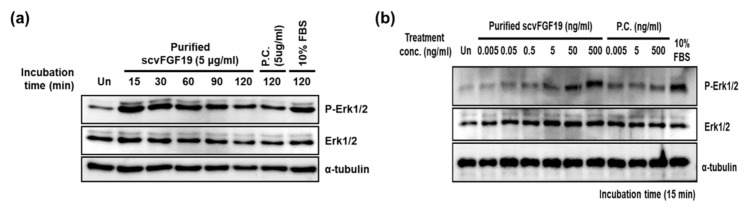
Biological activity of purified codon variant 19: (**a**) Time course of ERK1/2 kinase activation in HepG2 cells treated with 5 μg/mL of the codon variant 19. P.C.: positive control. (**b**) Dose-dependent ERK1/2 kinase activation in HepG2 cells treated with the defined amount of recombinant scvFGF19 (codon variant 19) for 15 min. All experiments were conducted at least three times according to the described procedure in the methods section.

It has been previously reported that hFGF19/FGFR4 signaling leads to the activation of Ras-Raf-Erk1/2 MAPK pathway, and thus functional assay for recombinant scvhFGF19 is conducted using HepG2 hepatocellular carcinoma cell line [8,15]. In this study, HepG2 cells were treated with purified scvhFGF19, and Erk1/2 phosphorylation in these cells was evaluated in a time- and dose-dependent manner. We found that scvhFGF19 induced a distinct phosphorylation of Erk1/2, and its effect decreased in a time-dependent manner (Figure 6a). In addition, scvhFGF19 activated Erk1/2 phosphorylation in a dose-dependent manner, similar to the commercially available positive control (Figure 6b). Altogether, this study demonstrated that the screened recombinant scvhFGF19 has the ability to phosphorylate Erk1/2 through a signaling pathway.

## 4. Discussion

hFGF19 plays pivotal functions in bile acid synthesis, energy metabolism, insulin sensitivity, liver regeneration, and anti-inflammation, although the underlying mechanisms are yet to be clarified [16,17]. Consequently, hFGF19 has become an attractive therapeutic drug for the treatment of obesity, fatty liver disease, and type 2 diabetes [18]. However, the production of hFGF19 without any modifications, especially in its amino acid sequence, has not been previously achieved in *E. coli.*

To express hFGF19 without fusion tags, we randomly substituted codons encoding the first 10 amino acids, except ATG, of hFGF19 with synonymous codons and screened variants expressing functional hFGF19 based on the fluorescence intensity of C-terminal-fused mCherry as a reporter. However, all the screened hFGF19 codon variants were expressed as insoluble aggregates. Moreover, after the centrifugation of the total cell lysate to remove insoluble aggregates, both the soluble and insoluble fractions emitted red fluorescence under UV light (data not shown). Red fluorescence emission in the soluble fraction indicated the presence of soluble scvhFGF19-mCherry and/or mCherry cleaved from scvhFGF19 by certain proteinases after or during translation. The cleavage site of mCherry is probably located in the long, disordered region of the C-terminus of hFGF19 [3], which is highly sensitive to proteolytic activity. In contrast, red fluorescence in the insoluble fraction is apparently emitted by the active inclusion bodies of mCherry [19]. Therefore, mCherry is not a suitable folding reporter for screening proteins with long, disordered regions at the C-termini, although it indicates increased expression levels of the target protein, as shown in this report. 

The aggregate-prone property of the scvhFGF19 screened in this study was alleviated by modulation of the cytoplasmic reducing environment using the alternative host *E. coli* Origami 2 instead of *E. coli* XL1-Blue and the co-expression of DsbC. This strategy involved the co-expression of DsbC and scvhFGF19 under three different conditions: under the control of a single promoter, under the control of two identical promoters, and under the control of two different promoters. We found that the use of different promoters for the expression of each gene could increase both DsbC and scvhFGF19 expression. Although the two different promoters are recognized by different RNA polymerases, viz. *E. coli* and T7 phage RNA polymerase, both genes were reproducibly expressed in this study. This may be due to the non-interference of the RNA polymerases with each other. Stochastic interaction between T7 RNA polymerase and two adjacent T7 promoters might underlie the low level of gene expression and reproducibility when two identical promoters were used. 

In a previous report, Kong et al. [8] showed that the solubility of TrxB-fused hFGF19 in the reducing environment of the cytoplasm could be improved by co-expression of DsbC alone, although the functional separation of hFGF19 from TrxB was not successfully achieved. In our study, co-expression of DsbC improved the solubility of scvhFGF19 in the non-reducing cytoplasmic environment of the usual *E. coli* host XL1-Blue. DsbC expression upstream, rather than downstream, of scvhFGF19 was more effective. However, despite an increased expression of DsbC, a major proportion of the expressed scvhFGF19 remained insoluble. These results indicated that the soluble overexpression of scvhFGF19 with two disulfide bonds was not innately difficult or prone to degradation by proteases, as shown in the results of the western blot analysis (truncated bands, just below the intact band of total cell lysate fraction; Figure 4 and Figure 5). It has been previously reported that high expression of DsbC can cause oxidation and exceed the reductive capacity of the cytoplasm [13]. Therefore, overexpression of DsbC alone was not sufficient to enhance the soluble expression of hFGF19, although we obtained approximately 5–7 mg of purified protein without any tag or additional amino acids using translational rate-controlled codon variants. Translational rate control promotes the interaction of a de novo synthetic protein with chaperones or exploits a fitness landscape for correct folding. 

The FGF19 subfamily, including 19, 21, and 23, shares a common core of around 140 amino acids with a high degree of sequence homology, and thus is expected to have similar structural properties. More intriguingly, the fold of hFGF19, including the predicted second disulfide bond, is similar to those of other FGFs, such as FGF8, 15, 17, 18, 21, and 23 [3,20]. Previous studies support that the FGF19 production strategy used in this study could be applied to express other related FGFs.

## Figures and Tables

**Table 1:** List of primers used in this study.

Primers	Sequence (5′→3′)	Cloning ^1^
pSCT5_FGF19-mC-fw	ATAACTAGTATGCGCCCCCTCGCCTTC	*Spe*Ⅰ
pSCT5_scvFGF19-mC-fw	ATAACTAGTATGCGNCCNCTNGCNTTYTCNGAYGCNGGNCCNCACGTGCACT	*Spe*Ⅰ
pSCT5_FGF19-mC-rv	ATAGGATCCCTTCTCAAAGCTGGGACTCCTCAC	*Bam*HⅠ
pSCT5_scvFGF19-rv	ATAAAGCTTTTACTTCTCAAAGCTGGGACTCCTC	*Hind*Ⅲ
pSCT5_ΔssDsbC-fw	ATAACTAGTATGGATGACGCGGCAATTC	*Spe*Ⅰ
pSCT5_ΔssDsbC-rv	ATAGGATCCTTATTTACCGCTGGTCATTTTTTGGTG	*Bam*HⅠ
pSCT5_FGF19/ΔssDsbC-fw	ATAGGATCCTAAATGGATGACGCGGCAATTCAAC	*Bam*HⅠ
pSCT5_FGF19/ΔssDsbC-rv	ATAAAGCTTTTATTTACCGCTGGTCATTTTTTGGTGTTC	*Hind*Ⅲ
pSCT5_scvFGF19-fw	ATACAATTTCACACAGAATTCATTAAAGAGGAGAAAGGATCCATGCG	*Bam*HⅠ
pSCT5_scvFGF19-rv	ATAAAGCTTTTACTTCTCAAAGCTGGGACTCCTC	*Hind*Ⅲ
pACYCDuet1_ΔssDsbC-fw	AGGAGATATACCATGGATGACGCGGCAATTCAACAAACG	*Nco*Ⅰ
pACYCDuet1_ΔssDsbC-rv	ATAGGATCCTTATTTACCGCTGGTCATTTTTTGGTGTTC	*Bam*HⅠ
pACYCDuet1_scvFGF19-fw	CAATTTCACACAGAATTCATTAAAGAGGAGAAACATATGCG	*Nde*Ⅰ
pACYCDuet1_scvFGF19-rv	ATACTCGAGTTACTTCTCAAAGCTGGGACTCCTCACG	*Xho*Ⅰ
pQE80L V-fw	AGTTAATTTCTCCTCTTTAATGAATTCTGTGTG	Infusion
pQE80L V-rv	CCGCCCTCTAGATTACGTGC	Infusion
pQHDuet_InfuΔssDsbC-fw	GAGGAGAAATTAACTATGGATGACGCGGCAATTC	Infusion
pQHDuet_InfuFGF19-rv	TAATCTAGAGGGCGGTTTTTAAGGCAGTTATTGGTGCCC	Infusion

^1^ Cloning of PCR product was conducted by specific digestion with restriction enzymes or homologous recombination. Underlined sequence indicates restriction sites.

## Supplementary Materials

The following are available online at https://www.mdpi.com/2076-2607/8/12/1942/s1.
