# Supplementary material for: Soluble Expression of hFGF19 without Fusion Protein through Synonymous Codon Substitutions and DsbC Co-Expression in E. coli
Source: Microorganisms. 2020 Dec 7;8(12):1942. doi: 10.3390/microorganisms8121942 (PMC7762406; doi:10.3390/microorganisms8121942)
Supplement: Supplementary file 1 [file microorganisms-08-01942-s001.pdf]

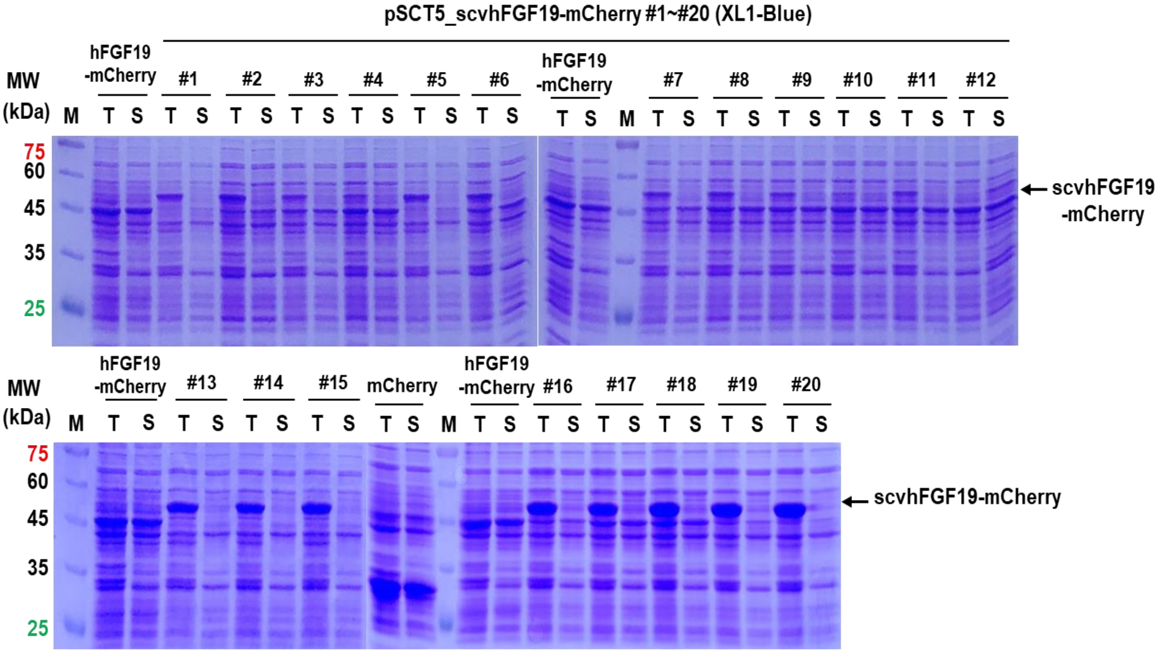

Figure S1. Twenty scvhFGF19-mCherry codon variants with improved expression levels were screened based on the enhanced fluorescence of red fluorescent mCherry and subjected to quantitative analyses of the expression patterns by SDS-PAGE (12% gel). The resulting gel was stained with Coomassie Brilliant Blue R250. #16, #17, #18, #19, and #20 mCherry-fused variants had higher expression levels and soluble fraction ratios than others, and thus selected for further experiments. MW: protein size marker; mCherry: expressed mCherry as a control; hFGF19: mCherry-fused wild type hFGF19; 1–20: primarily screened codon variants scvhFGF19-mCherry.

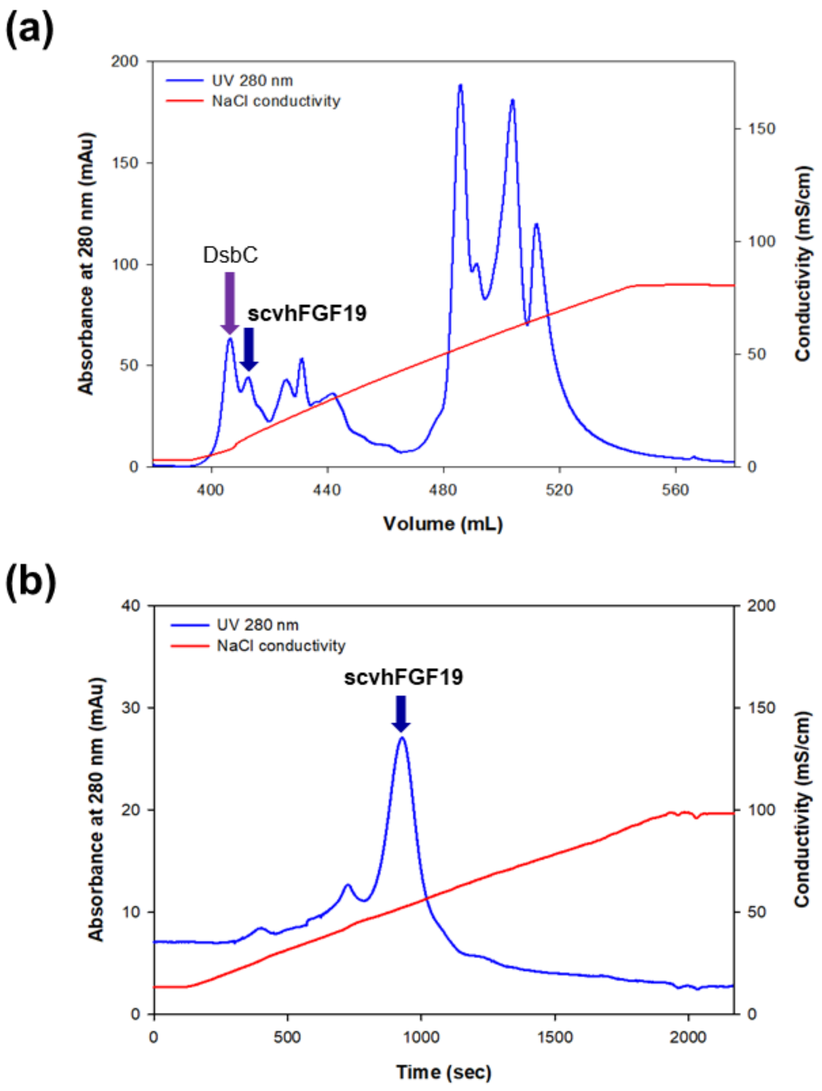

Figure S2. Elution profiles of anion-exchange (**a**) and heparin affinity chromatography (**b**) during the purification of scvhFGF19. Repeated experiment showed the identical elution patterns.
